# Surgery for Deeply Located Hydatid Cysts of the Liver: A Simple Alternative

**DOI:** 10.1155/2000/36518

**Published:** 2000-01

**Authors:** Kirien T. Kjossev, Julian E. Losanoff

**Affiliations:** 1 Department of General Surgery Military Medical Academy Sofia Bulgaria; 2 Department of Emergency Surgery Military Medical Academy Sofia Bulgaria; 3 P.O. Box 159 Sofia 1606 Bulgaria

## Abstract

Gaining access to deeply located hydatid cysts of the liver using conventional surgical techniques may be accompanied by significant intra- and postoperative complications. In addition, obliteration of the cyst cavity is still a matter of controversy. We developed a novel method for easy access to deep hydatid cysts using a water jet dissector (Parenchimotom 01, TOSA, Pleven, Bulgaria). At pressure of 20 Bar using a 0.2mm nozzle, a corridor is created through the liver parenchyma overlying the cyst; vessels and biliary duct are thus clearly displayed as linear structures traversing the corridor and are ligated and divided under direct visual control. The fibrous capsule of the cyst is spared by the jet. Following endocystectomy performed in the ordinary manner, the cyst cavity is filled with gelatin sponge; a passive tube drain is placed in contact with the liver incision.

In allowing for a selective dissection of the liver parenchyma, the jet makes safe access to deeply located hydatid cysts possible. On the other hand, the gelatin sponge induces good fibroblast response and assists in rapid and effective obliteration of the residual cavity. This novel technique works well in our hands but more extensive studies are necessary before its final acceptance.


*HPB Surgery*, 2000, Vol. 11, pp. 307–310
Reprints available directly from the publisher
Photocopying permitted by license only

© 2000 OPA (Overseas Publishers Association) N.V.
Published by license under
the Harwood Academic Publishers imprint,
part of The Gordon and Breach Publishing Group.
Printed in Malaysia.

### Surgery for Deeply Located Hydatid Cysts of the Liver: A Simple Alternative

KIRIEN T. KJOSSEV[a,*] and JULIAN E. LOSANOFF[b]

[a] *Department of General Surgery,* [b] *Department of Emergency Surgery, Military Medical Academy, Sofia, Bulgaria*

*(Received 3 June 1999)*

**Gaining access to deeply located hydatid cysts of the liver using conventional surgical techniques may be accompanied by significant intra- and postoperative complications. In addition, obliteration of the cyst cavity is still a matter of controversy. We developed a novel method for easy access to deep hydatid cysts using a water jet dissector (Parenchimotom 01, TOSA, Pleven, Bulgaria). At pressure of 20 Bar using a 0.2 mm nozzle, a corridor is created through the liver parenchyma overlying the cyst; vessels and biliary duct are thus clearly displayed as linear structures traversing the corridor and are ligated and divided under direct visual control. The fibrous capsule of the cyst is spared by the jet. Following endocystectomy performed in the ordinary manner, the cyst cavity is filled with gelatin sponge; a passive tube drain is placed in contact with the liver incision.**

**In allowing for a selective dissection of the liver parenchyma, the jet makes safe access to deeply located hydatid cysts possible. On the other hand, the gelatin sponge induces good fibroblast response and assists in rapid and effective obliteration of the residual cavity. This novel technique works well in our hands but more extensive studies are necessary before its final acceptance.**

*Keywords:* Hydatid cysts, access, liver, water jet dissection, selective dissection, endocystectomy, cyst cavity obliteration, gelatin sponge

### INTRODUCTION

The most frequent location of hydatid disease is the liver and unfortunately, surgery may be accompanied by significant intra- and postoperative complications, thus making hydatidosis a serious problem. A variety of surgical approaches have been proposed depending on both the site and size of the cyst, with varying degree of success.

Superficial cysts, even if located over the right posterolateral aspect of the liver, can easily be managed by one of the standard techniques including cystectomy or pericystectomy [1]. On the other hand, deeply located hydatid cysts are usually adherent to the hepatic hilum or the retrohepatic Vena cava and may thus seem "untouchable" to the surgeon because of the existing high risk of damage to the major vascular and biliary structures. In such circumstances, safe removal of the cyst may require significant operative trauma such as

*Address for correspondence: P.O. Box 159, 1606 Sofia, Bulgaria, e-mail: freckles@public.digsys.bg thoracophrenolaparotomy [2]. Obliteration of the cyst cavity itself may prove even more hazardous.

We present a simple procedure for endocystectomy by use of a water-jet dissector and employment of hemostatic sponge to promote safe obliteration of the cyst cavity.

### TECHNIQUE

The abdomen is entered through midline or right subcostal laparotomy depending on surgeon's own preference. Following visual inspection of the liver for evaluation of the size, site, and extent of the cyst, intraoperative ultrasound examination is performed to identify the thinnest layer of hepatic parenchyma overlying the cyst. To prevent any possible spillage of cyst contents, packs soaked in 20% saline are placed around the planned line of liver parenchymal incision. The liver capsule is cut with a knife. Following this, "corridoring" of the liver parenchyma overlying the cyst is performed by the water jet (Parenchimotom 01, TOSA, Pleven, Bulgaria) at pressure of 20 Bar using a 0.2 mm nozzle. Liver parenchymal cells are shed off by the jet in a linear fashion, thus, a corridor measuring approximately 2.0/2.0 cm is created through the organ. In this way, vessels and biliary ducts become visible as linear structures traversing the corridor; under visual control, they are easily clamped, divided, and ligated. The pericyst is exposed in a relatively bloodless plane, the cyst is punctured, and opened following instillation of traditional scolicidal agents such as 20% saline. Before opening of the cyst cavity, scolocidal-soaked packs may be sutured to the margins of the liver corridor to again prevent spillage of cyst contents. The pericyst is then sharply cut and any cyst contents is evacuated. The cavity is inspected and cystobiliary communications, if present, are closed. If possible, the pericyst is sutured to the liver capsule using several single

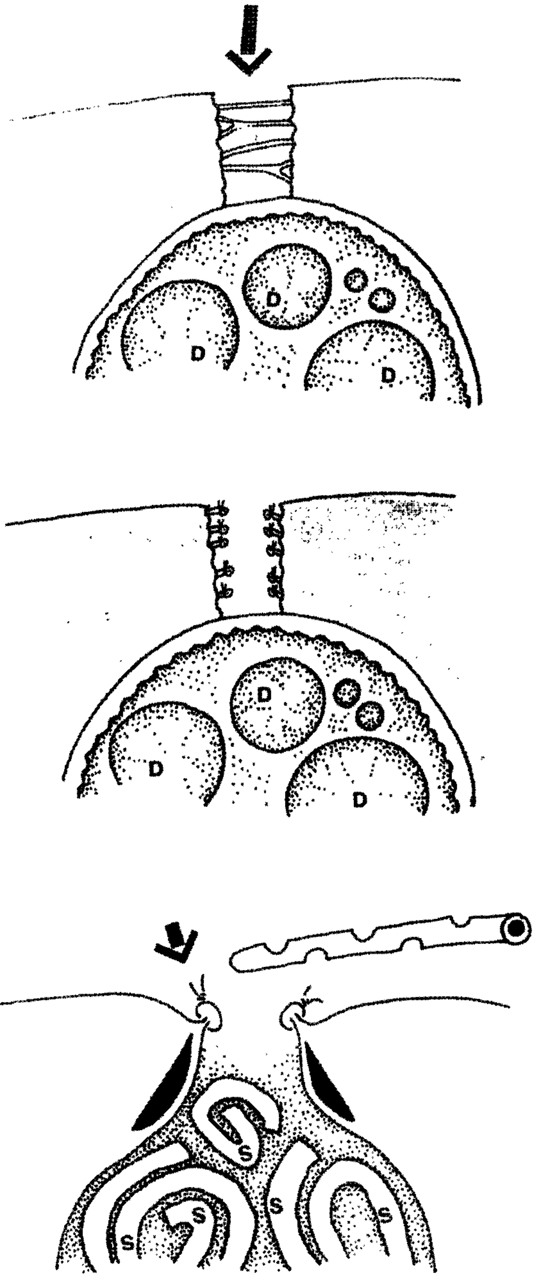

FIGURE 1 Cross-sectional diagrammatic drawing illustrating: *top*- the corridor through the hepatic parenchyma overlying the cyst (arrow) with vessels and biliary duct left intact and traversing the line of dissection; *middle*- all vessels and biliary ducts ligated separately under direct vision, and *bottom*- gelatin-sponge-filled cyst cavity with fibrous capsule sutured to the hepatic capsule (arrow), and a drain contacting the hepatic incision. D- daughter cysts; S- gelatin sponge.

stitches to aid in achieving safer hemostasis. The residual cavity is tightly filled with gelatin sponge (Gelaspon, Chauvin Ankerpharm GmBH, Rudolstadt, Germany) and is left unclosed (Fig. 1). A passive silicon tube drain is placed to contact the liver incision and is exteriorized dependently and laterally. Abdominal wall closure is routine.

### DISCUSSION

The above described technique focuses on two new aspects of surgery for liver hydatidosis. First, to our knowledge this is the first application of water-jet technology to liver hydatid cysts. Water jet dissection was introduced in clinical practice in 1982 for the treatment of metastatic malignancies of the liver [3]. This technology has been used successfully in both open and laparoscopic liver surgery. It has been widely investigated and its own advantages and disadvantages are well-known [4]. At our Department, the water-jet apparatus became part of the standard operative theater equipment since 1992 and was extensively used in selected cases of liver surgery. By being a tissue-selective type of dissection, water jet dissection was shown to spare fibrotic structures such as vessels and bile ducts while disintegrating liver parenchymal tissue at pressure of 20 Bar using a nozzle of 0.2 mm [4]. In this context, the jet was shown to spare the fibrotic capsule of hydatid cysts, thus making safe access to the cyst possible. The risk of cyst contents spillage was eliminated as a result. It must be noted however, that at least theoretically, another type of tissue selective dissection using ultrasonic power (Cavitron Ultrasonic Surgical Aspirator, CUSA) may be used for gaining access to deeply located hydatid disease of the liver. Surprisingly, we were unable to identify any other articles from the literature dealing with the application of selective types of dissection for accessing deeply located hydatid cysts.

Second, the management of the residual cyst cavity is still subject of controversy. Several methods have gained popularity including external tube drainage, deroofing, capitonnage, introflexion, omentoplasty, or simple cyst closure [1]. The application of each method is strongly dictated by the size and location of the cyst; if the latter is located deeply in the hepatic parenchyma, only two safe alternatives exist: external tube drainage or primary closure. In our experience unfortunately, these have frequently been associated with prolonged biliary leakage, infection of the residual cavity, biloma formation, and resulting prolonged hospital stay. In the present technique, absorbable gelatin sponge was used to promote rapid obliteration of the residual cavity. Its clinical use as topical hemostatic agent has been controversial because of the fact that this type of sponge is the least effective one in achieving hemostasis [5]. However, the use of gelatin sponge in our technique was deemed appropriate because of its property of inducing good granulation with subsequent fibrosis [5]; moreover, this sponge is completely absorbable and is thus less prone in acting as a nidus of infection. The gelatin sponge is inexpensive, too, and is videly available.

In conclusion, we present a novel technique which proved simple and effective in our hands. Further larger prospective studies are needed to compare its effectiveness with other well-established techniques for management of deep liver hydatid cysts.

### *References*

[1] Ariogul, O., Emre, A., Alper, A. and Uras, A. (1989). Introflexion as a method of surgical treatment for hydatid disease. *Surgery, Gynaecology & Obstetrics*, **169**, 356–358.

[2] Gonzalez, E. M., Selas, P. R., Martinez, B., Garcia, I. G., Carazo, F. P. and Pascual, M. H. (1991). Results of surgical treatment of hepatic hydatidosis: Current therapeutic modifications. *World Journal of Surgery*, **15**, 254–263.

[3] Papachristou, D. N. and Barters, R. (1982). Resection of the liver with a water jet. *British Journal of Surgery*, **69**, 93–94.

[4] Penchev, R. D., Kjossev, K. T. and Losanoff, J. E. (1997). Application of a new water jet apparatus in open hepatobiliary surgery: Hepatic resection, cholecystectomy, common bile duct lavage. *International Surgery*, **82**, 182–186.

[5] Coln, D., Horton, J., Ogden, M. E. and Buja, L. M. (1983). Evaluation of hemostatic agents in experimental splenic lacerations. *American Journal of Surgery*, **145**, 256–259.

